# Risk prediction in stable coronary artery disease using quantified myocardial ischemia and necrosis by cardiac magnetic resonance imaging - a prospective long-term follow-up trial

**DOI:** 10.1186/1532-429X-18-S1-O58

**Published:** 2016-01-27

**Authors:** Dominik Buckert, Nils Dyckmanns, Wolfgang Rottbauer, Peter Bernhardt

**Affiliations:** Innere Medizin II, Uniklinik Ulm, Ulm, Germany

## Background

Until now, no consensus is reached for the standardized assessment and quantification of extent and severity of myocardial ischemia and necrosis by cardiac magnetic resonance imaging (CMR). However, there is a good evidence base suggesting that these parameters might ameliorate established risk prediction models and thus might lead to an improved management of patients with stable coronary artery disease (CAD). Objective of this study therefore is to elaborate an easy-to-use algorithm for the quantification of ischemia and necrosis and to validate the obtained data in a large cohort of CAD patients.

## Methods

Patients with known or suspected CAD referred for adenosine-perfusion CMR were consecutively and prospectively enrolled. Examinations were conducted on a 1.5 Tesla whole-body scanner (Intera, Philips Medical Systems, Best, the Netherlands) using a 5-element phased array surface receiver coil. All patients underwent standard first-pass perfusion imaging (balanced fast-field echo sequence, repetition time 2.6 ms, echo time 1.3 ms, saturate pre-pulse with 100-ms delay, flip angle 50°, 40 dynamics, voxel size 2.8 × 2.9 mm, slice thickness 10 mm) under adenosine infusion for the detection of myocardial ischemia and late gadolinium enhancement (LGE) imaging (inversion-recovery gradient-echo sequence (repetition time 7.1 ms, echo time 3.2 ms, voxel size 1.6 × 1.6 mm, slice thickness 8 mm) for the assessment of myocardial necrosis. Based on these sequences, ischemia and necrosis were quantified using a novel algorithm.

Primary endpoint was defined as combination of cardiac death, non-fatal myocardial infarction and stroke. Several risk prediction models containing clinical and CMR parameters were built and analyzed with regard to correct risk stratification and endpoint prediction.

## Results

The study cohort consisted of 845 patients. Median follow-up was 3.66 [1.48; 7.27] years. During this time, 61 primary endpoints occurred. Cutoff values for myocardial ischemia and LGE could be defined (for ischemia: ≥6% of left ventricular mass, HR: 4.03, p: < .0001; for LGE: >33% of left ventricular mass, HR: 9.50, p: < .0001). A risk prediction model containing this information proved to be superior in comparison to models based on conventional CMR features (increase in χ²: from 45.004 to 61.545, integrated discrimination index: .04074, p: .011, net reclassification index: .11793, p: .035).

## Conclusions

Myocardial ischemia and necrosis above the defined cutoff values are strongly associated with major clinical endpoints. Quantification of these parameters provides additive information and leads to improved risk stratification.

